# Prolonged morphine administration alters protein expression in the rat myocardium

**DOI:** 10.1186/1423-0127-18-89

**Published:** 2011-11-30

**Authors:** Zdenka Drastichova, Jitka Skrabalova, Jan Neckar, Frantisek Kolar, Jiri Novotny

**Affiliations:** 1 Department of Physiology, Faculty of Science, Charles University, Prague 2; 2 Department of Developmental Cardiology, Institute of Physiology, Academy of Sciences of the Czech Republic; 3 Centre for Cardiovascular Research, Prague 4, Czech Republic

**Keywords:** rat myocardium, morphine, proteomics

## Abstract

**Background:**

Morphine is used in clinical practice as a highly effective painkiller as well as the drug of choice for treatment of certain heart diseases. However, there is lack of information about its effect on protein expression in the heart. Therefore, here we aimed to identify the presumed alterations in rat myocardial protein levels after prolonged morphine treatment.

**Methods:**

Morphine was administered to adult male Wistar rats in high doses (10 mg/kg per day) for 10 days. Proteins from the plasma membrane- and mitochondria-enriched fractions or cytosolic proteins isolated from left ventricles were run on 2D gel electrophoresis, scanned and quantified with specific software to reveal differentially expressed proteins.

**Results:**

Nine proteins were found to show markedly altered expression levels in samples from morphine-treaded rats and these proteins were identified by mass spectrometric analysis. They belong to different cell pathways including signaling, cytoprotective, and structural elements.

**Conclusions:**

The present identification of several important myocardial proteins altered by prolonged morphine treatment points to global effects of this drug on heart tissue. These findings represent an initial step toward a more complex view on the action of morphine on the heart.

## Background

Morphine, a highly effective analgesic used for treatment of different kinds of chronic pain states, may also exert significant cardiovascular effects [1-3]. Moreover, under certain conditions, this drug can be also implicated in the acquisition of cardioprotection against ischaemia-reperfusion injury [4-6]. However, the molecular mechanisms and consequences of morphine actions on the heart have not yet been fully elucidated. The majority of studies dealing with morphine in the field of cardiology are oriented on clinical usage of this drug and current cardiovascular research has been limited to the evaluation of factors or pathways believed to contribute to its physiological action, such as δ- and κ-opioid receptors, cyclooxygenase-2, inducible nitric oxide synthase or reactive oxygen species [7-10]. Because of great importance of this drug in clinical practice, and in view of morphine unwanted effects on one side and cardioprotective potential on the other, investigation of its impact on the heart at the molecular level deserves more attention. A better understanding of the molecular mechanisms involved in morphine actions will require the evaluation of corresponding protein translation and the integration of these findings into the overall context.

Proteomic technology allows for the examination of global alterations in protein expression, and can provide new insights into the cellular mechanisms involved in cardiac dysfunction or other diseased states [11,12]. Apparently, this approach may also help to uncover the possible broader effects of variable exogenous or endogenous stimuli on the heart muscle. Proteomics is therefore very useful to investigate the changes in protein expression induced by various drugs or medicaments. To date, several reports have been published about the influence of morphine on protein expression in brain tissue [13-15], but there is lack of information regarding cardiac proteins under these conditions. Therefore, the main aim of this study was to assess the presumed effect of prolonged treatment of rats with high dose morphine on myocardial protein expression. Application of differential proteomic analysis enabled us to identify nine markedly altered proteins of different types in myocardial preparations from morphine-treated rats.

## Methods

### Materials

Acrylamide and bis-acrylamide were from SERVA (Heidelberg, Germany), SYPRO Ruby stain from Molecular Probes (Eugene, OR, USA) and nitrocellulose membrane from Schleicher-Schuell (Erdmannhausen, Germany). Immobiline DryStrips, Pharmalyte buffer, and secondary anti-rabbit antibody labeled with horseradish peroxidase were from GE Healthcare (Piscataway, NJ, USA) and rabbit primary antibodies were purchased from Santa Cruz Biotechnology (Santa Cruz, CA, USA). All other chemicals were from Sigma (St. Louis, MI, USA) and they were of the highest purity available.

### Animals and morphine treatment

Adult male Wistar rats were housed in groups of 3-4 in Plexiglas chambers with food and water available *ad libitum *and maintained on a 12-h light/dark cycle. All animal experiments were conducted in accordance with the Guide for the Care and Use of Laboratory Animals as adopted by the National Institutes of Health (NIH Publication No. 85-23, revised 1996). The rats were injected intramuscularly with saline (CON group; *n *= 10) or morphine (10 mg/kg per day) (MOR group; *n *= 10) for 10 consecutive days. In some experiments, naloxone (10 mg/kg per day) or a combination of naloxone and morphine (both 10 mg/kg per day) were administered. Twenty four hours after the last dose, the animals were sacrificed by cervical dislocation and the hearts rapidly excised, dissected, snap frozen in liquid nitrogen and stored at -80°C until use. In the withdrawal experiments, samples of cardiac tissue were procured from the animals sacrificed 3 days after the last dose of morphine.

### Cardiac tissue fractionation

Frozen samples of left ventricles were placed into 10 volumes of ice-cold TMES buffer (20 mM Tris, 3 mM MgCl_2_, 1 mM EDTA and 0.25 M sucrose; pH 7.4) containing the protease inhibitor cocktail (Roche Diagnostics), cut in small pieces and homogenized in an Ultra-Turrax blender (15 s). The resulting suspension was further homogenized for 1 min in a glass homogenizer with a motor-driven Teflon pestle, and then centrifuged at 600 × g for 10 min at 4°C in order to remove large tissue debris and nuclear fragments. The resulting postnuclear supernatant (PNS) was applied on the top of a 18% Percoll solution in TMES buffer and centrifuged at 60, 000 × g for 15 min. In this way, two layers containing opaque material were separated on the gradient. The translucent top portion (~ 3 ml) of the gradient containing soluble material was further centrifuged at 300, 000 × g to yield a clear supernatant cytosol fraction. The upper layer enriched in plasma membranes and the lower layer rich in mitochondria were separately diluted in TME buffer and centrifuged at 150, 000 × g for 1 h. The plasma membrane and mitochondrial pellet was then resuspended in TME buffer, snap-frozen in liquid nitrogen and stored in aliquots at -80°C until use.

### Electrophoresis and immunoblotting

Protein samples solubilized in Laemmli buffer were resolved on standard 10% polyacrylamide gels and transferred to nitrocellulose membrane. After blocking with 3% nonfat dry milk, the membranes were probed with the appropriate antibodies and the immunodecorated protein bands visualized by enhanced chemiluminiscence technique according to the manufacture's instructions (Pierce Biotechnology, Rockford, IL, USA). The immunoblots were scanned and quantitatively analyzed by ImageQuant™ TL software (Amersham Biosciences).

### Proteomic analysis

Samples of all three fractions were precipitated with 6% trichloracetic acid for 1.5 h on ice and then solubilized in IEF buffer containing 7 M urea, 2 M thiourea, 4% CHAPS, 1% DTT, 1% ampholines pH 3-10 and 0.01% bromphenol blue. Samples of 100 μg protein or 3 mg protein (for MALDI-TOF MS/MS analysis) were applied on immobilized pH 4-7 linear gradient strips. Proteins were separated using the Multiphor II apparatus (150 V, 5 h; 500 V, 1 h; 3500 V, 12.5 h). Strips were equilibrated for 15 min in 4 ml of EQ buffer (50 mM Tris, pH 6.8, 6 M urea, 30% glycerol, 2% SDS) containing 1% DTT, followed by a second 15-min equilibration in EQ buffer containing 4% iodacetamide and separated in the second dimension on 10% SDS-polyacrylamide gels using the Hoefer apparatus. Gels were stained with the fluorescent dye SYPRO Ruby or colloidal Coomassie G-250 for MALDI-TOF MS/MS and spot density was quantitatively analyzed by PDQuest software (Bio-Rad, Hercules, CA, USA) as described previously [16]. Proteins with altered expression were identified using a 4800 Plus MALDI TOF/TOF Analyzer [16]. The obtained MS spectra were searched against the rat IPI database by Mascot v. 2.1 software (Matrix Science Ltd., London, UK). Criteria for positive identification of proteins with MS were set according to the scoring algorithm delineated in Mascot http://www.matrixscience.com. A minimum protein score of 60 (p < 0.05) were considered as statistical values for identification.

### Miscellaneous

Protein concentration was determined by the BCA method [17]. Master reference gels for 2D proteome analysis were established by averaging the data of 12 replicas of each experimental group. Proteins isolated from spots in 2D gels showing significant quantitative differences (at least a 2-fold difference) between the groups were subjected to mass spectrometric analysis.

## Results and discussion

Although a series of proteins in the heart have been shown to be qualitatively or quantitatively dysregulated under various pathophysiological conditions, there is no information about cardiac protein expression following morphine administration. Therefore, this study was designed to assess the presumed effect of prolonged morphine treatment of rats on protein profiling in cardiac tissue using a proteomics-based approach. The hearts obtained from control and morphine-treated (10 mg/kg per day) animals did not differ in weight and did not show any visual signs of gross pathology.

### 2D gel separation of myocardial proteins

**Figure 1:**
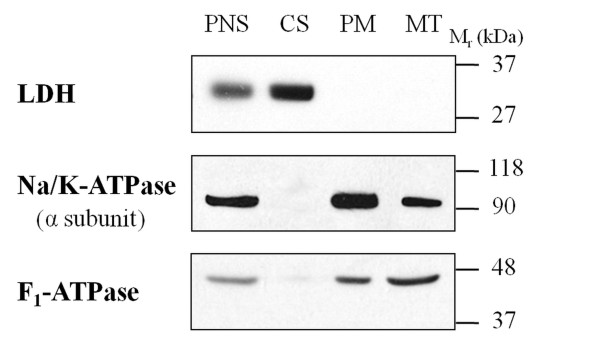
**Distribution of marker proteins in fractionated samples of cardiac tissue**. Protein samples (30 μg) of the postnuclear supernatant (PNS), cytosolic (CS), plasma membrane-enriched (PM) and mitochondrial (MT) fractions were subjected to immunoblotting with antibodies against lactate dehydrogenase (LDH), the α subunit of Na, K-ATPase, and F_1_-ATPase.

Subcellular fractionation of tissue samples represents an important first step prior to 2D gel-based proteomics experiments, because it may reduce a high complexity of these samples and improve detection of low abundance proteins [18]. Therefore, left ventricles were homogenized and subfractionated into a plasma membrane-enriched fraction (PM), mitochondrial fraction (MT) and cytosol (CS). To examine the specificity of fractionation of heart tissue, immunoblot analysis was carried out against known subcellular markers. Na, K-ATPase (marker of PM), F_1_-ATPase (marker of MT), and lactate dehydrogenase (marker of CS) were chosen because of their well established roles in the respective cellular fractions and immunoblots demonstrated the appropriate subcellular location for these proteins (Figure 1). Whereas there was no apparent cross-contamination of the CS fraction with membrane-bound proteins, the PM fraction was partially contaminated with MT proteins and vice versa. Nevertheless, this partial cross-contamination did not hamper subsequent proteomic analyses.

**Figure 2:**
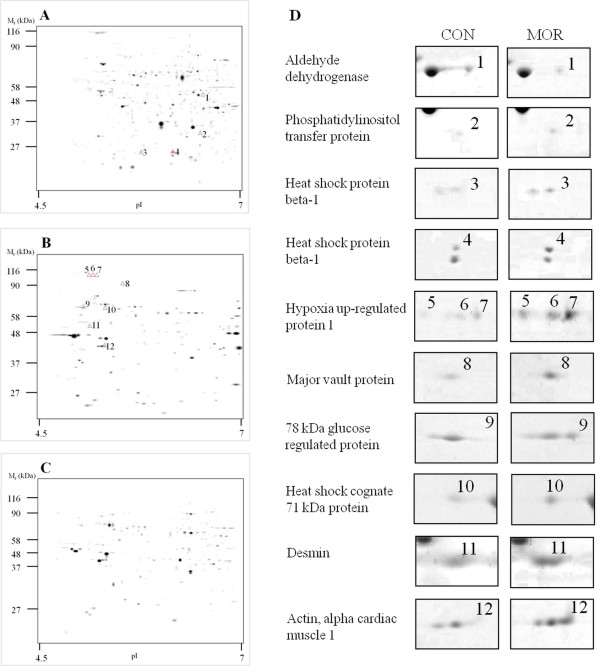
**Two-dimensional protein profiles of myocardial tissue**. Representative gel images of the cytosolic (A), plasma membrane-enriched (B) and mitochondrial (C) fractions stained with SYPRO Ruby are displayed. The positions of molecular weight markers are indicated to the left and the p*I *at the bottom of each panel. The protein spots with markedly different densities in myocardial preparations from control and morphine-treated rats are numbered from 1 to 12 and the respective portions of 2D gels are shown in greater magnification in panel D. The corresponding identifications are listed in Table 1. Two spots were detected for heat shock protein beta-1 (No. 3 and 4) and three spots for hypoxia up-regulated protein 1 (No. 5-7), possibly due to post-translational modifications.

All three fractions (PM, MT and CS) were resolved in pH 4-7 IPG strips (IEF) and 10% polyacrylamide gels (SDS-PAGE). After SYPRO Ruby staining, about 500 protein spots were detected on each two-dimensional gel. The marked differences in 2D gel electrophoretic spot patterns observed between the CS, PM and MT fractions indicated that the procedure used for cardiac tissue fractionation was good enough to separate different cellular compartments (Figure 2A, B, and 2C). Our next comparative image analysis of 2D maps prepared from cardiac fractions of morphine-treated and control rats revealed 12 differentially expressed protein spots (Figure 2D). Only spots which had over two-fold changes in density were classified as down- or up-regulated. The average difference in spot densities within control groups was about 20%. Two-fold changes in density can thus be considered sufficient to determine altered proteins. Whereas 4 of these significantly altered spots were found in the CS fractions, the other 8 were in the PM fractions. Interestingly, no significant changes were observed in 2D maps prepared from the MT fractions. All the altered proteins were subsequently identified by mass spectrometry.

### Identification of differentially expressed proteins

Protein spots showing altered abundance levels were excised from the gel, in-gel digested with trypsin, and analyzed by MALDI-TOF MS/MS as described previously [16]. Peptide mass-fingerprinting and subsequent database searching revealed the identity of altered proteins as summarized in Table 1. As can be seen from the table, only one of all the identified proteins was down-regulated (mitochondrial precursor of aldehyde dehydrogenase) while all the others were up-regulated to different extent. Two of the altered proteins (heat shock protein beta-1 and hypoxia up-regulated protein 1) occured with multiple forms differing in gel mobility. This likely reflects different post-translational modifications of these proteins, which have been previously described [19,20].

**Table 1:** List of cardiac proteins with significantly altered expression after prolonged morphine treatment

**Spot No**.	Protein name	Protein ID	p*I*	MW (kDa)	Protscore	Expression (fold change)
1	Aldehyde dehydrogenase, mitochondrial precursor	ALDH2_RAT	6.63	56.5	74	↓ 3.3
2	Phosphatidylinositol transfer protein, alpha isoform	PIPNA_RAT	5.96	31.9	189	↑ 2.3
3	Heat shock protein beta-1	HSPB1_RAT	6.12	22.9	152	↑ 3.5
4	Heat shock protein beta-1	HSPB1_RAT	6.12	22.9	373	↑ 5.2
5	Hypoxia up-regulated protein 1	HYOU1_RAT	5.11	111.3	69	↑ 3.1
6	Hypoxia up-regulated protein 1	HYOU1_RAT	5.11	111.3	88	↑ 3.3
7	Hypoxia up-regulated protein 1	HYOU1_RAT	5.11	111.3	65	↑ 3.1
8	Major vault protein	MVP_RAT	5.42	95.8	66	↑ 5.3
9	78 kDa glucose-regulated protein	GRP78_RAT	5.07	72.3	95	↑ 3.2
10	Heat shock cognate 71 kDa protein	HSP7C_RAT	5.37	70.9	59	↑ 2.3
11	Desmin	DESM_RAT	5.21	53.5	391	↑ 4.3
12	Actin, alpha cardiac muscle 1	ACTC_RAT	5.23	42.0	160	↑ 2.6

### The role of differentially expressed proteins

Aldehyde dehydrogenases (ALDHs) are important enzymes that eliminate toxic aldehydes by catalyzing their oxidation to non-reactive acids and they may have a role in combating oxidative stress by reducing the cellular 'aldehydic load' [21]. However, Wang et al. [22] recently reported that the level of ADH 2 is significantly down-regulated under oxidative stress conditions. Our present finding of decreased level of mitochondrial precursor of ADH in myocardial preparations from morphine-treated rats conforms well to the latter observation, because it is known that chronic exposure to morphine can induce oxidative stress in various tissues [23,24].

Phosphatidylinositol transfer proteins (PITPs) are responsible for the transport of phosphatidylinositol and other phospholipids between membranes, and they participate in many cellular processes including signaling, lipid metabolism and membrane traffic [25]. It might be speculated that the increased level of the α isoform of PITP in morphine-exposed hearts can perhaps promote cardiomyocyte survival especially by affecting the aforementioned signaling processes. Phosphatidylinositol transfer activities of PITPs have been shown to be required for both PLC- and PI3K-mediated signaling [26,27], and the activity of these two signaling pathways is thought to play an important role in the development of a cardioprotective phenotype [28,29]. It might be worth mentioning in this context that morphine has been reported as an agent capable to induce cardioprotection under certain conditions [4-6].

**Figure 3:**
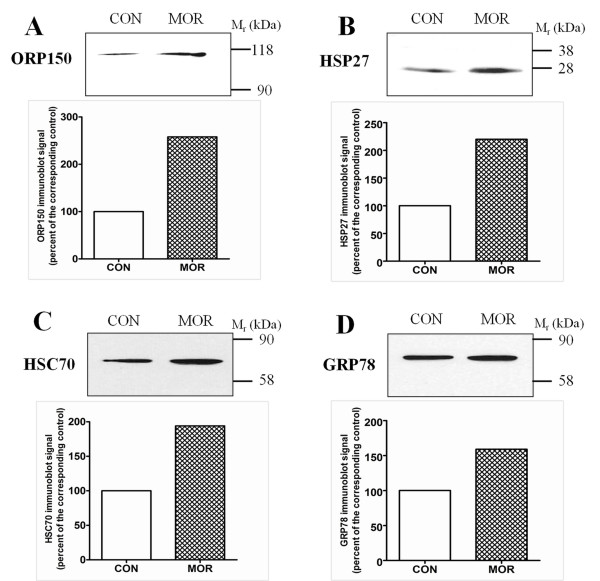
**Immunoblot analysis of cytoprotective proteins in myocardial preparations from rats affected by morphine exposure**. Samples from control (CON) and morphine-treated (MOR) rats were resolved by SDS-PAGE, transferred to nitrocellulose membrane and probed with indicated antibodies. All lanes were loaded with 50 μg proteins. The relative levels of ORP150, hypoxia up-regulated protein 1 (A), HSP27, heat shock protein beta-1 (B), HSC70, heat shock cognate 71 kDa protein (C) and GRP78, 78 kDa glucose-regulated protein (C) were assessed by scanning densitometry.

Nevertheless, a major role in cytoprotection can be attributed to the set of heat shock proteins (HSPs), which function to limit protein aggregation, facilitate protein refolding and chaperone other proteins [30]. HSP27 (heat shock protein beta-1) and HSC70 (heat shock cognate 71 kDa protein) can be up-regulated under oxidative stress conditions [31,32], and, intriguingly, the levels of both these proteins were significantly elevated in cardiac preparations from morphine-treated rats. These data comply with the notion that morphine can cause oxidative stress. In line with this are also the observed increased levels of ORP150 (hypoxia up-regulated protein 1) and GRP78 (78 kDa glucose-regulated protein), two chaperone molecules previously shown to be up-regulated by endoplasmic reticulum stress [20,33]. These two proteins serve important cytoprotective functions and can be up-regulated after numerous cellular insults. The increased expression of all the above cytoprotective proteins after prolonged morphine treatment was confirmed by immunoblot analysis (Figure 3). These results suggest that morphine administration can trigger stress response in cardiomyocytes and that the levels of intracellular heat shock and other stress-related proteins increase in order to provide cellular protection and maintain homeostasis under these conditions.

Changes found in the expression of two cytoskeletal/contractile proteins, desmin and cardiac muscle alpha actin 1, may imply that prolonged morphine treatment could modulate formation of muscle fibres and muscle contraction. Previous data indicated that the cytoskeleton, together with contractile proteins, is a major site of the cellular damage and impairment of myocyte function in heart failure, and significant increase in these proteins, including desmin and alpha-cardiac actin, has been described in myocyte hypertrophy and failing hearts [34,35]. These proteins play important roles in cardiac integrity and contractility, but the consequences of their up-regulation are not quite clear.

The major vault protein (MVP) is supposed to be involved in different cellular processes, including intracellular signaling, cell survival, and differentiation and it can play a protective role against some xenobiotics and other stresses [36]. Our present finding of increased expression of this protein after morphine exposure may thus suggest that MVP could participate in mediating the presumed cardioprotective effects of this drug. Recently published data by Ikeda et al. [36] indicate that the PI3K/Akt pathway might be implicated in the cytoprotective effect of MVP. Interestingly, the protective function of PI3K signaling could be synergistically enhanced also by the observed increase of PITP expression.

### Verification of the specificity of morphine effects

**Figure 4:**
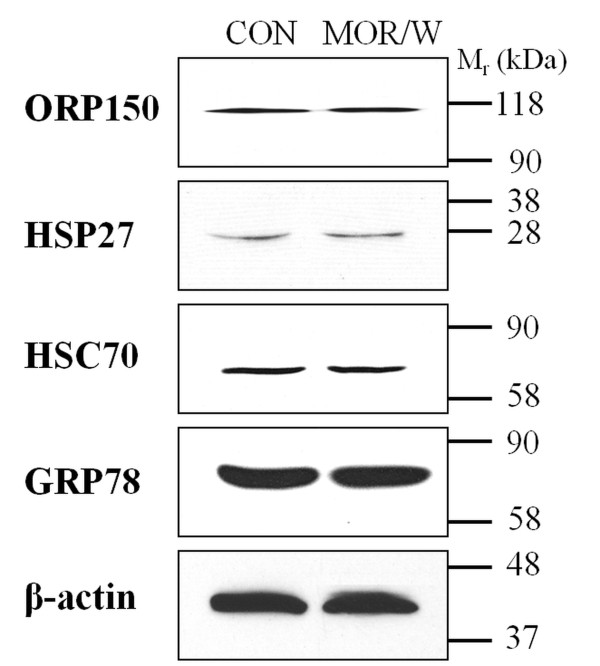
**Effect of morphine withdrawal on the levels of cytoprotective proteins**. Myocardial preparations (50 μg proteins) from control rats (CON) and morphine-treated (MOR/W) rats kept drug-free for 3 days after the last dose were resolved by SDS-PAGE, transferred to nitrocellulose membrane and probed with indicated antibodies. β-Actin was used as an internal standard.

To determine whether the alterations observed in some cytoprotective proteins (ORP150, HSP27, HSC70 and GRP78) were induced by morphine or drug withdrawal, a set of experiments was performed using a group of animals, which were treated with morphine (10 mg/kg per day) for 10 days and then withdrawn for 3 days. As shown in Figure 4, the 3-day morphine withdrawal resulted in normalization of protein expression to control levels. These results indicate that the increase in the expression of cytoprotective proteins induced by morphine exposure can be reversed by the drug withdrawal.

**Figure 5:**
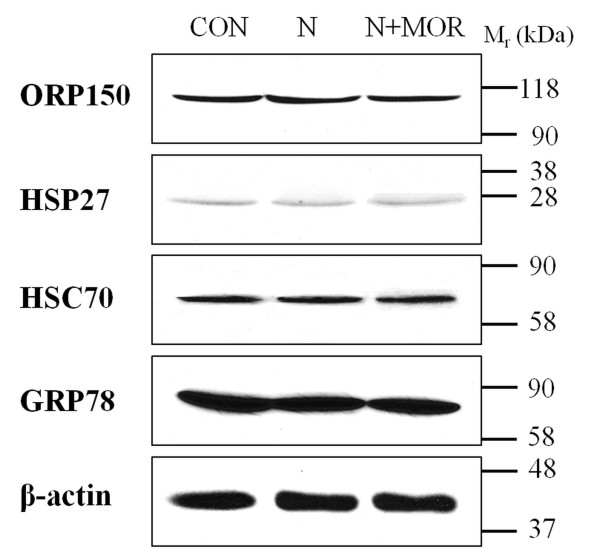
**Effect of naloxone or a combination of naloxone and morphine on the levels of cytoprotective proteins**. Myocardial preparations (50 μg proteins) from control (CON), naloxone-treated (N) or treated with both naloxone and morphine (N+MOR) rats were resolved by SDS-PAGE, transferred to nitrocellulose membrane and probed with indicated antibodies. β-Actin was used as an internal standard.

To exclude the possibility that the observed protein changes could have been mediated by signaling pathways independent of opioid receptor activation, the opioid antagonist naloxone was employed in subsequent experiments [37]. Naloxone alone or a combination of naloxone and morphine was administered to rats and the levels of selected cytoprotective proteins in myocardial preparations were assessed by immunoblotting (Figure 5). Naloxone did not alter expression of these proteins and the effect of morphine was completely blocked by this compound. These observations allow us to conclude that morphine exerted its effects through the activation of opioid receptors and its action was specific.

### Concluding remarks

In this study, we adopted a proteomics-based approach to investigate the effect of morphine exposure on cardiac protein expression. Our results demonstrate that 10-day high dose morphine treatment altered the expression of several important proteins that are involved in anti-oxidative defence or cytoprotection, signaling, and cytoskeletal/contractile structures. These data indicate for the first time that morphine can induce a wide range of effects in the heart and future studies will involve exploring the function and regulation of these proteins in more detail.

## Competing interests

The authors declare that they have no competing interests.

## Authors' contributions

ZD and JS performed the major experiments and analyzed the data. JNe made substantial contribution to conception and design of the study and participated in experiments with animals and in data interpretation. FK participated in data interpretation and manuscript improvement. JNo conceived the study, designed the experiments, and wrote the manuscript. All authors read and approved the final manuscript.
